# MiR-3180 inhibits hepatocellular carcinoma growth and metastasis by targeting lipid synthesis and uptake

**DOI:** 10.1186/s12935-023-02915-9

**Published:** 2023-04-11

**Authors:** Jie Hong, Jie Liu, Yanan Zhang, Lihua Ding, Qinong Ye

**Affiliations:** 1 Medical School of Guizhou University, Guiyang, China; 2 Department of Cell Engineering, Beijing Institute of Biotechnology, Beijing, China

**Keywords:** Lipid synthesis, Lipid transport, miR-3180, SCD1, CD36

## Abstract

**Purpose:**

Reprogrammed lipid metabolism is a hallmark of cancer that provides energy, materials, and signaling molecules for rapid cancer cell growth. Cancer cells acquire fatty acids primarily through de novo synthesis and uptake. Targeting altered lipid metabolic pathways is a promising anticancer strategy. However, their regulators have not been fully investigated, especially those targeting both synthesis and uptake.

**Methods:**

Immunohistochemistry was performed on samples from patients with hepatocellular carcinoma (HCC) to establish the correlation between miR-3180, stearoyl-CoA desaturase-1 (SCD1), and CD36 expression, quantified via qRT-PCR and western blotting. The correlation was analyzed using a luciferase reporter assay. Cell proliferation, migration, and invasion were analyzed using CCK-8, wound healing, and transwell assays, respectively. Oil Red O staining and flow cytometry were used to detect lipids. Triglycerides and cholesterol levels were analyzed using a reagent test kit. CY3-labeled oleic acid transport was analyzed using an oleic acid transport assay. Tumor growth and metastasis were detected in vivo in a xenograft mouse model.

**Results:**

MiR-3180 suppressed de novo fatty acid synthesis and uptake by targeting the key lipid synthesis enzyme SCD1 and key lipid transporter CD36. MiR-3180 suppressed HCC cell proliferation, migration, and invasion in an SCD1- and CD36-dependent manner in vitro. The mouse model demonstrated that miR-3180 inhibits HCC tumor growth and metastasis by inhibiting SCD1- and CD36-mediated de novo fatty acid synthesis and uptake. MiR-3180 expression was downregulated in HCC tissues and negatively correlated with SCD1 and CD36 levels. Patients with high miR-3180 levels showed better prognosis than those with low levels.

**Conclusions:**

Our investigation indicates that miR-3180 is a critical regulator involved in de novo fatty acid synthesis and uptake, which inhibits HCC tumor growth and metastasis by suppressing SCD1 and CD36. Therefore, miR-3180 is a novel therapeutic target and prognostic indicator for patients with HCC.

**Supplementary Information:**

The online version contains supplementary material available at 10.1186/s12935-023-02915-9.

## Introduction

Cancer is defined as a systematic metabolic dysfunction disease [1]. Lipid metabolism is activated and significantly upregulated during cancer development and progression, providing energy and material for cancer cell proliferation and metastasis [2]. Therefore, reprogrammed lipid metabolism is seen as a new hallmark of cancer malignancy, and targeting this pathway has become a promising cancer therapeutic strategy [3–5].

Cancer cells obtain lipids mainly through de novo lipid synthesis and uptake, which are both activated [6, 7]. Lipids are sequentially synthesized by a series of enzymes. ATP citrate lyase (ACLY) converts citrate into acetyl-CoA, whose conversion to malonyl-CoA is catalyzed by acetyl-CoA carboxylase (ACC), and fatty acid synthase (FASN) catalyzes palmitate synthesis from malonyl-CoA. Stearoyl-CoA desaturase-1 (SCD1) produces monounsaturated fatty acids from saturated fatty acids. In addition to de novo lipid synthesis, fatty acid uptake from the exogenous environment is essential in cancer cells. Lipid uptake occurs mainly through the membrane glycoprotein CD36, which binds fatty acids and transports them into the cells [8]. Several proteins regulate the lipid metabolism pathway. For instance, SREBP is a direct master regulator of lipid synthesis enzyme expression, including ACLY, ACC1, and FASN, which thereby enhances de novo lipid synthesis. The Myc oncoprotein can synergistically enhance lipid synthesis via SREBP [9]. Liver X receptor, pregnane X receptor, and peroxisome proliferator-activated receptor γ regulate CD36 [10]. Overexpression of enzymes and regulators involved in the lipid metabolic pathway has been reported in cancer, correlating with the clinical outcomes of cancer patients. ACLY is overexpressed in patients with hepatocellular carcinoma (HCC), indicating shorter overall survival (OS), compared with low ACLY levels [11, 12]. SB-204990, an ACLY inhibitor, has shown anticancer effects in vivo [5, 7]. FASN is highly expressed in lung cancer, and its expression is negatively correlated with the clinical outcomes of HCC patients [13]. The FASN inhibitor TVB-2640 has been used to treat non-small cell lung cancer and colon cancer in clinical studies [14]. SCD1 inhibition prevents HCC [15]. Furthermore, anti-CD36 antibodies have shown a significant antimetastatic effect in oral cancer xenograft models [16]. SREBP1 overexpression has been observed in various human cancers, and its specific inhibitor 25-hydroxycholesterol suppresses cancer cell migration and proliferation [6, 17]. However, inhibitors targeting lipid synthesis or uptake alone cannot fully inhibit cancer progression. As cancer cells acquire fatty acids primarily through de novo synthesis and uptake, targeting both lipid synthesis and uptake is a promising anticancer strategy. However, the regulatory factors targeting both lipid synthesis and uptake have not been clearly studied. Therefore, it is urgent to explore these regulators.

MiRNAs are small non-coding RNAs containing approximately 22 nucleotides that regulate gene expression, and they have been reported as key lipid metabolism regulators. MiR-4310 inhibits lipid synthesis by suppressing FASN and SCD1, thus suppressing HCC growth and metastasis [18]. MiR-192-5p regulates lipid synthesis in nonalcoholic fatty liver disease through SCD-1 [19]. MiR-195 inhibits proliferation, invasion, and metastasis in breast cancer cells by targeting FASN, HMGCR, ACACA, and CYP27B1 [20]. MiR-758-5p regulates cholesterol uptake by targeting the CD36 3’-UTR [21]. MiR-9 directly targets the 3’-UTR of cholesterol acyltransferase, an enzyme that converts cholesterol into cholesterol esters for storage along with triglycerides in the cores of cytosolic lipid droplets [22]. However, miRNAs that target both lipid synthesis and transport pathways have not been investigated.

In this study, we found that miR-3180 inhibits both de novo lipid synthesis and uptake by targeting SCD1 and CD36. Further investigation indicated that miR-3180 inhibits HCC cell proliferation, migration, invasion, and metastasis in vitro and in vivo in a lipid synthesis- and uptake-dependent manner. Therefore, miR-3180 plays an important role in HCC development and progression and may be a promising target for HCC therapy.

## Methods

### Cell lines and cell culture

Human HCC cell lines MHCC-97H, HepG2, and HEK293T were obtained from the American Type Culture Collection. These cells were tested for mycoplasma contamination and cultured in Dulbecco’s modified Eagle medium (DMEM, Gibco) supplemented with 10% fetal bovine serum (TIANHANG) and 1% penicillin–streptomycin solution (100 ×) (Thermo Fisher Scientific) in humidified air with 5% CO_2_ at 37 °C.

### Plasmids, lentiviruses, and reagents

The SCD1 and CD36 coding regions were amplified using PCR and cloned into the pCDH vector (System Biosciences). SCD1 and CD36 shRNA sequences (Table S1) were cloned into pSIH1-H1-Puro vector (System Biosciences). To obtain lentiviruses, the constructed lentiviral vector and pPACK Packing Plasmid Mix (System Biosciences) were co-transfected into HEK-293T cells in the presence of polybrene (10 μg/ml). Lentivirus-infected HCC cell lines were prepared by screening with puromycin (1 µg/ml) for 7–10 days. MiR-3180-5p mimics, inhibitors, and negative controls were obtained from Jintuosi (Beijing, China, Table S1). Polyethylenimine (Polysciences) and Lipofectamine RNAiMAX (Invitrogen) were used to transfect plasmids and miRNAs, respectively, according to the manufacturer’s instructions. β-Actin antibodies (sc-47778) and anti-mouse horseradish peroxidase (HRP)-conjugated IgG (sc-2748) were purchased from Santa Cruz Biotechnology. Specific antibodies against SCD1 (AF7944) and anti-CD36 (AF6414) were purchased from Beyotime Biotechnology.

### Immunoblotting

Cells were collected and lysed in RIPA lysis buffer containing protease inhibitors for 30 min on ice. After evaluating protein concentrations using a BCA protein assay kit (Thermo Fisher Scientific), equivalent proteins were separated via SDS–PAGE and transferred to a nitrocellulose membrane. To block nonspecific reactions, the membrane was deposited in 5% skimmed milk and incubated with SCD1 and CD36 primary antibodies (both 1:500) at 4 °C for 12 h. The membrane was incubated with anti-rabbit HRP-conjugated IgG for 2 h at room temperature. The bands were visualized using enhanced chemiluminescence (Thermo Fisher Scientific).

### Reverse transcription-quantitative PCR (RT–qPCR)

Total RNA was extracted from the cells using TRIzol reagent according to the manufacturer’s instructions (Invitrogen). Equal amounts of RNA were reverse-transcribed into cDNA. For miR-3180-5p, reverse transcription was performed using the miRcute miRNA First-Strand cDNA Synthesis Kit protocol (Tiangen). RT-qPCR was performed using a miScript SYBR Green PCR Kit (Qiagen) in a Bio-Rad CFX96 system. The expression data were analyzed using the 2^−ΔΔCt^ method and normalized to β-actin. The primer sequences are listed in Table S2.

### Luciferase reporter assay

Wild type (WT) and mutant (Mut) predicted 3′-UTR binding sequences of miR-3180 in CD36 and SCD1 were obtained using PCR and cloned into pmir-GLO dual-luciferase miRNA target expression vectors (Promega). The primer sequences of the WT and Mut 3’-UTRs are listed in Table S3. WT and Mut 3′-UTR reporters were co-transfected with miR-3180 mimics or a negative control. The transfected cells were collected, and luciferase reporter analyses were performed according to the manufacturer’s instructions (Promega).

### CCK-8 assay

Cell proliferation assays were performed according to the CCK-8 manufacturer’s instructions (Dojindo Laboratories). Briefly, 2 × 10^3^ cells were plated in 96-well plates in triplicate. The proliferation assay was performed every 24 h using a CCK-8 kit with an OD450 microplate reader.

### Wound healing assay

A wound healing assay was performed to detect cell migration. Cells were cultured to 80–90% cell density in 6-well plates and scratched with a 200 μl pipette tip. The scratched wells were cultured for 12 h in serum-free DM.

EM. The cells were photographed at 0 and 16 h at the same location. ImageJ software was used to determine the distance between the migrating cells.

### Transwell assay

A total of 5 × 10^4^ transfected cells were plated into the upper chamber of a 24-well transwell chamber (Corning Costar) and covered with a matrix (200 µL per well). After culturing for 12 h in serum-free medium, the chamber was removed from the plate. The chamber was then fixed with 4% paraformaldehyde and stained with 0.5% crystal violet for 30 min. The invading cells were photographed using a microscope and analyzed using ImageJ.

### Oil Red O staining

The cells were plated and cultured to 70% density on coverslips placed in 24-well plates. The cells were fixed with 4% paraformaldehyde for 30 min, washed twice for 5 min with PBS, and stained with Oil Red O (Solarbio) working solution (stock solution/ddH2O, 3:2) for 10 min. Nuclei were stained using Harris. The stained cells were photographed and analyzed using the ImageJ software.

### Flow cytometry

The transfected cells were stained with BODIPY 493/503 (1 μg/ml) for 20 min according to the manufacturer’s instructions. The stained cells were separated and analyzed using flow cytometry (BD Biosciences). The data were analyzed using FlowJo V10 software.

### Triglyceride and cholesterol assay

A total of 3000 cells were collected and lysed according to the manufacturer’s instructions (Puilai, Beijing, China). Triglyceride and cholesterol levels were detected using histological triglyceride and total cholesterol enzyme assay kits, respectively.

### Oleic acid transport assay

Transfected HCC cells were cultured on coverslips and stained with CY3-labeled oleic acid (2.5 μM in BSA) for 24 h. The cells on the coverslips were fixed with 4% paraformaldehyde for 20 min. After washing twice with PBS, the cells were treated with BODIPY 493/503 (1 μg/ml) for 20 min and DAPI (5 μg/ml) for 2 min. Images were acquired using a fluorescence microscope.

### Tumor growth and metastasis assay in nude mice

All animal experiments were approved by the Institutional Animal Care Committee of the Beijing Institute of Biotechnology. Six-week-old male nude mice were purchased from Sibeifu (Beijing, China). For tumor growth analysis, 1 × 10^7^ cells were injected subcutaneously into BALB/c nude mice (six groups, n = 6 per group) to develop tumor xenografts. Tumor size was measured using calipers at the indicated times. Tumor volume was calculated using the following formula: (longest diameter × shortest diameter^2^)/2. Mice were grouped randomly when the tumor volume increased to 50 mm^3^ and intratumorally injected with miR-3180-3p antagomir or negative control antagomir (GenePharma) (15 μg in 100 μl PBS per mouse, twice a week, eight times total). To analyze lung metastasis, 1 × 10^6^ MHCC-97H cells were injected along with antagomiR-3180 (15 μg in 100 μl PBS) or negative control (15 μg in 100 μL PBS) into the tail vein of BALB/c nude mice (six groups, n = 6 per group). After 35 days of injection, all mice were euthanized, and the lungs were fixed for hematoxylin and eosin staining.

### MiRNA in situ hybridization (MISH) and immunohistochemical staining of clinical samples

HCC tumor tissues were collected from the Chinese PLA General Hospital with approval from its Institutional Review Committee, and the patients provided informed consent. HCC patient diagnosis was confirmed by histological or at least two imaging examinations and serum alpha-fetoprotein > 400 ng/ml. All cases used for the clinical relevance study included 58 males and 14 females with 35–65 years of age (mean age: 52.6 years). The follow-up time was 6.5–69.2 months (mean: 33.2 months).

To ascertain miR-3180 location and expression levels, MISH was performed with the specimen at 42 °C for 24 h using a miRNA probe according to the manufacturer’s instructions (EXON BIO, Guangzhou). Immunohistochemistry (IHC) with a TSA fluorescence system kit (APExBIO) was used to detect CD36 and SCD1 expression, according to the manufacturer’s protocol. Rabbit anti-CD36 (AF7944; Beyotime) and anti-SCD1 (AF5168; Beyotime) antibodies were diluted 1:100 for IHC. The miRNA, CD36, and SCD1 signals were amplified using the TSA Plus cyanine 5 system (K1052, APExBIO), cyanine 3 system (K1051, APExBIO), and FITC systems, respectively. The percentages of stained cells (0–100%) with staining intensities (low, 1%; medium, 2%; strong, 3%) were used to calculate the H scores. All optimal IHC values were scored and determined as follows: CD36, SCD, and miR-3180 scores < 1.6 were considered low. The optimal IHC score cut-off value was calculated using the receiver operating characteristic curve.

### Statistical analysis

All statistical analyses were performed using the GraphPad Prism 7 software. Statistical significance was set at *P* < 0.05. All experiments were performed at least three times. Student’s *t*-test and one-way ANOVA were used to compare the groups. Correlations between miR-3180, CD36, and SCD scores were assessed using Spearman rank correlation analysis.

## Results

### MiR-3180 negatively correlated with CD36 and SCD1 expression and predicted clinical outcomes in HCC patients

**Fig. 1:**
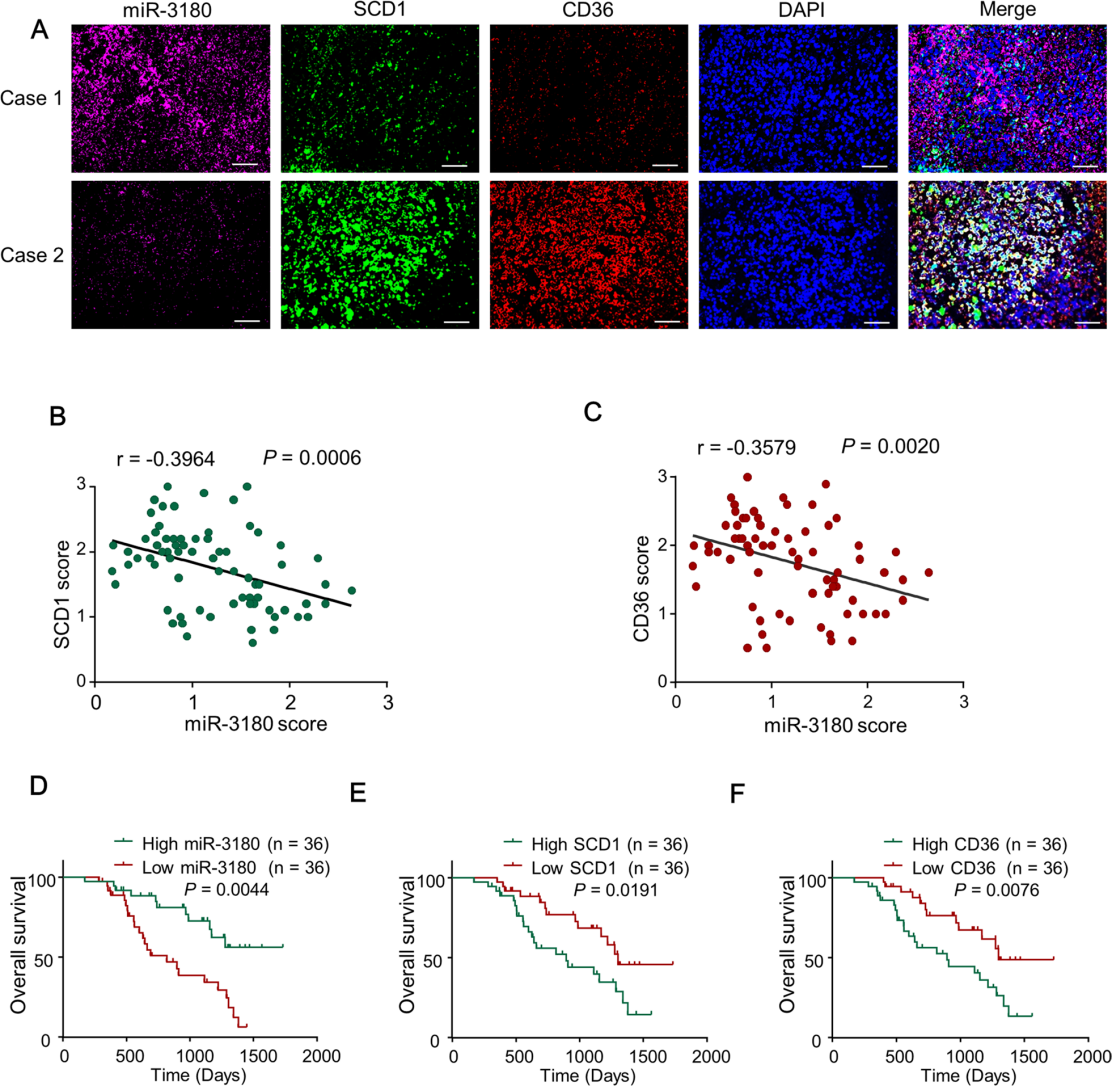
The expression of miR-3180 is negatively correlated with the expression of CD36 and SCD1 and predicts the clinical outcomes of HCC patients. **a** Representative images of triple-labeled miR-3180 (purple), SCD1 (green) and CD36 (red) in HCC patients. The nuclei were stained with DAPI (blue). Scale bar: 100 μm. **b, c** The relationships of the expression of miR-3180 and SCD1 or CD36 in the 72 patients with HCC were detected with Pearson’s correlation test. **d-f** The correlation analysis of the expression of miR-3180 (**d**), SCD1 (**e**) and CD36 (**f**) with the overall survival in 72 HCC patients with the Kaplan–Meier test

Using genome-wide CRISPR/Cas9-knockout library screening, we found that miR-3180 knockout greatly enhanced lipid content in HCC cells [18]. We further detected miR-3180-regulated genes involved in lipid metabolism signaling using the miRBase and miRWalk databases and found that miR-3180 targeted the fatty acid synthesis enzyme SCD1 and fatty acid translocase CD36. Next, we investigated the clinical significance of miR-3180 in HCC patients. MiR-3180 expression was inversely correlated with that of CD36 (r = − 0.3579, *P* = 0.0020) and SCD1 (r = − 0.3964, *P* = 0.0006) (Fig. 1A–C). Importantly, HCC patients with higher miR-3180 levels had longer OS, and those with lower CD36 and SCD1 expression had longer OS (Fig. 1D–F). These data suggest that the miR-3180-CD36/SCD1 axis is clinically significant and may regulate both lipid synthesis and uptake in HCC cells.

### MiR-3180 regulated lipid content by regulating both de novo synthesis and uptake

**Fig. 2:**
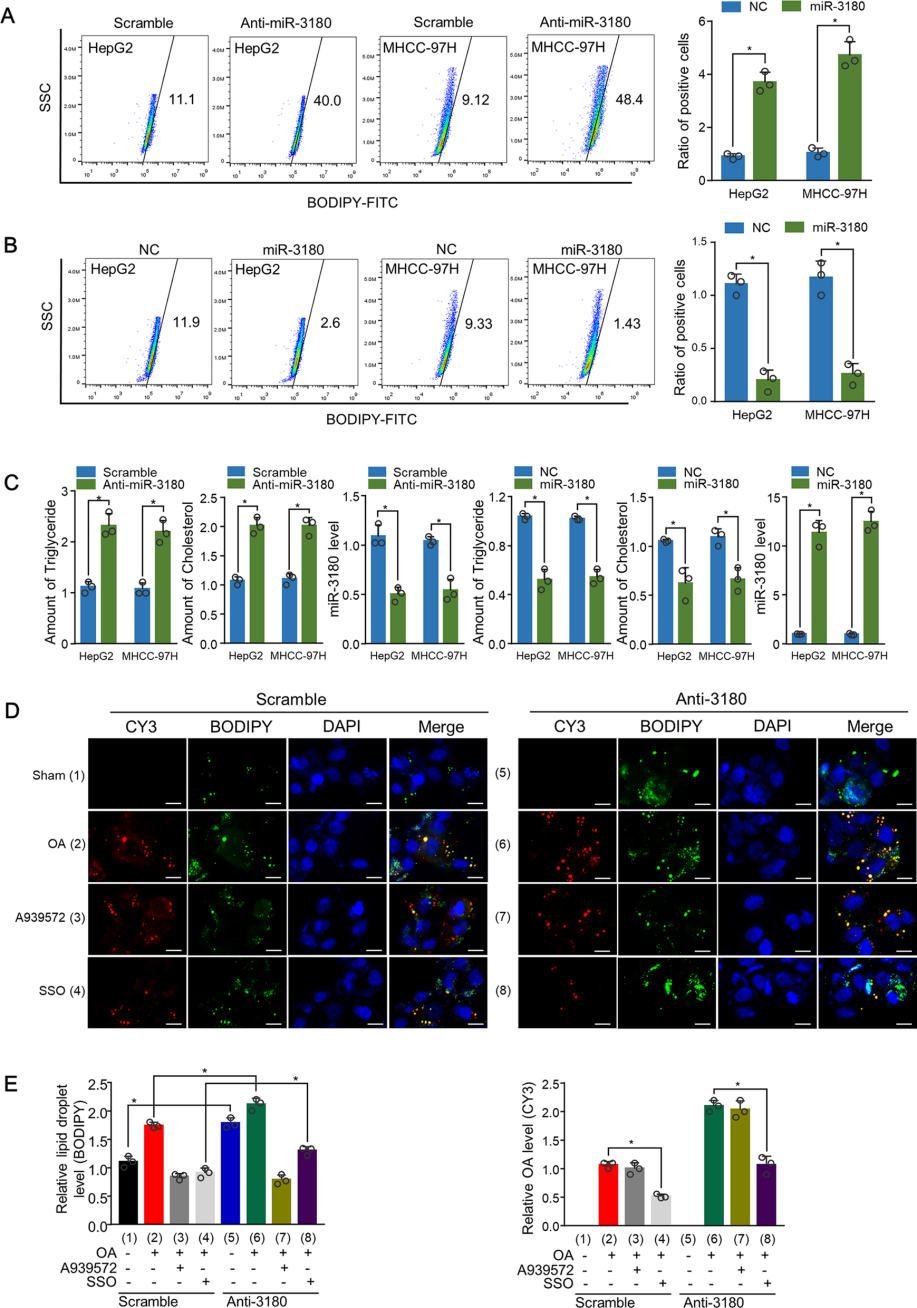
miR-3180 regulates lipid content in HCC cells. **a, b** Representative flow cytometry analysis of lipid content in HepG2 and MHCC-97H cells transfected with **a** scramble or miR-3180 inhibitors or **b** negative control (NC) or miR-3180 mimics as indicated. Statistical analysis of lipid content is displayed in the right panel. **c** Relative triglyceride and cholesterol levels in HepG2 and MHCC-97H cells transfected with miR-3180 or its inhibitor. The expression of miR-3180 is shown in the right panel. **d** Representative images of CY3-labeled oleic acid (OA) and BODIPY 493/503 in MHCC-97H cells treated with or without A939572 or sulfosuccinimidyl oleate sodium (SSO). Scale bar: 20 µm. **e** Statistical analysis of lipid content (left panel) and OA level (right panel) of (d). The data shown are presented as the mean ± SD of triplicate measurements with similar results. *p < 0.05, **p < 0.01

Given the clinical significance and correlation between miR-3180 and SCD1/CD36, we further investigated the effect of miR-3180 on the lipid content and triglyceride and cholesterol levels in HCC cells. The lipid content was upregulated in anti-miR-3180-transfected cells, whereas it significantly decreased in miR-3180-transfected cells (Fig. 2A, B). Furthermore, anti-miR-3180 increased triglyceride and cholesterol levels in HepG2 and MHCC-97H cells, while miR-3180 mimics inhibited their levels in HepG2 and MHCC-97H cells (Fig. 2C).

Since CD36 and SCD1 are predicted targets of miR-3180, we examined the effect of miR-3180 on lipid uptake and de novo synthesis. As CD36 imports oleic acid into cells, we traced CY3-labeled oleic acid (Fig. 2D, E). We found that the CD36 inhibitor sulfosuccinimidyl oleate sodium (SSO) inhibited OA uptake (CY3 of 4 vs*.* 1), and the SCD inhibitor (A939572) inhibited lipid content (BODIPY of 3 vs*.* 1), suggesting that these inhibitors are effective. Importantly, anti-miR-3180 enhanced OA uptake (CY3 of 6 vs*.* 2), and the CD36 inhibitor SSO abrogated anti-miR-3180-mediated OA uptake (CY3 of 8 vs. 6). Moreover, anti-miR-3180 promoted lipid content (BODIPY of 6 vs*.* 2), which the SCD inhibitor A939572 almost abolished (BODIPY of 8 vs*.* 6). Taken together, miR-3180 inhibits both de novo lipid synthesis and lipid uptake in HCC cells.

### MiR-3180 inhibited the 3’-UTR activity of CD36 and SCD1

**Fig. 3:**
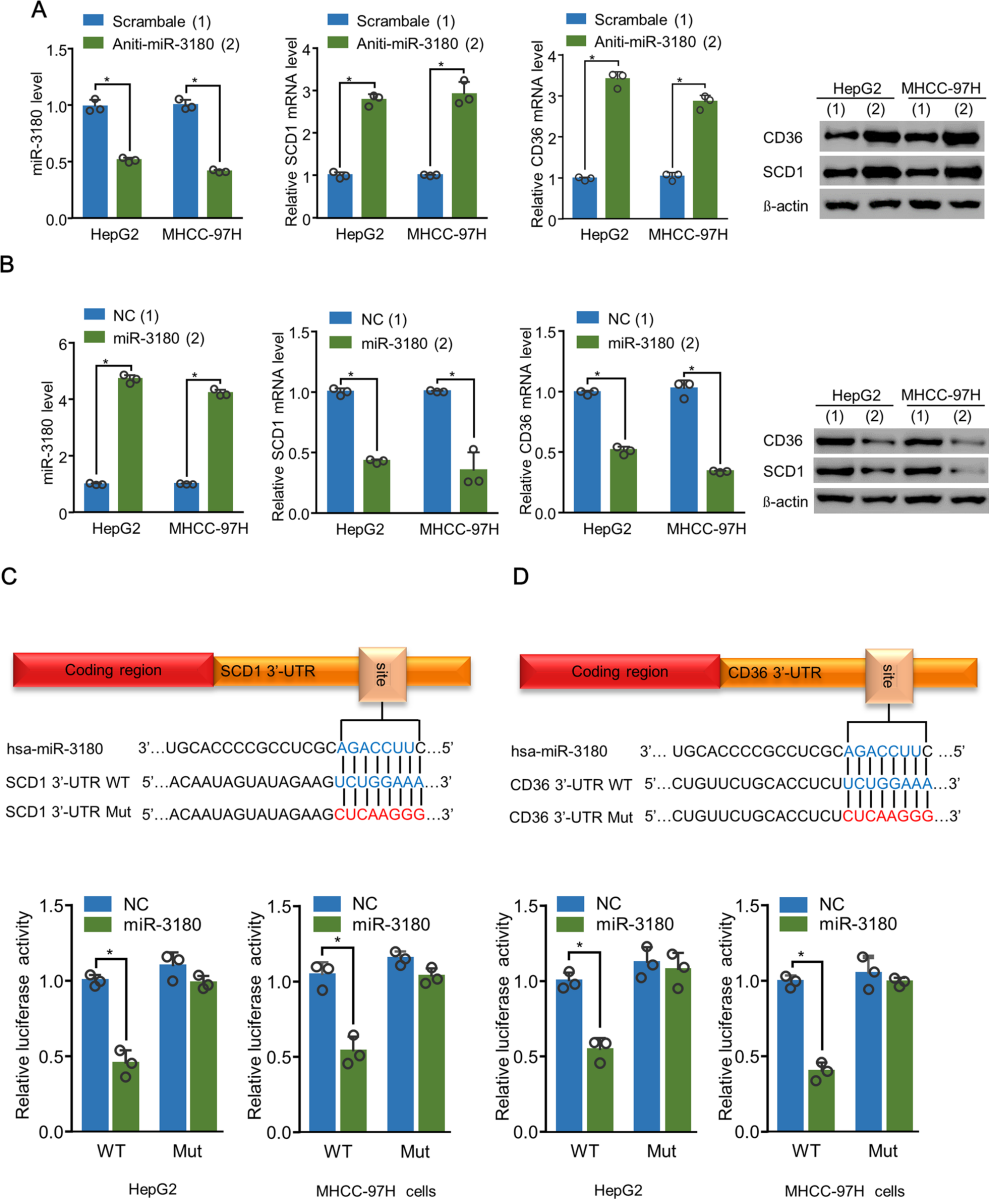
MiR-3180 inhibits the 3′-UTR activity of CD36 and SCD1. **a** The mRNA and protein expression of CD36 and SCD1 in HepG2 and MHCC-97H cells transfected with scramble or miR-3180 inhibitor using RT–qPCR and immunoblotting analysis. β-actin was used as a loading control for immunoblotting. **b** The mRNA and protein expression of CD36 and SCD1 in HepG2 and MHCC-97H cells transfected with NC or miR-3180 mimics using RT–qPCR and immunoblotting analysis. β-actin was used as a loading control for immunoblotting. **c, d** Luciferase reporter analysis of wild-type (WT) and mutated (Mut) 3′-UTR activity of SCD1 (c) or CD36 (d) in HepG2 and MHCC-97H cells. The putative miR-3180 target sequences in the CD36 and SCD1 3′-UTRs are shown in the top panel. The data shown are presented as the mean ± SD of triplicate measurements with similar results. **p < 0.01

Since miR-3180 expression correlated negatively with CD36 and SCD1 expression in patients with HCC, we explored whether miR-3180 targeted SCD1 and CD36. RT–qPCR and western blotting revealed that anti-miR-3180 upregulated SCD1 and CD36 mRNA and protein expression in HepG2 and MHCC-97H cells (Fig. 3A). In contrast, miR-3180 inhibited these expressions (Fig. 3B). To determine whether miR-3180 inhibits SCD1 and CD36 expression by targeting their 3’-UTR, we examined its effect on WT and Mut 3’-UTR activity. We found that miR-3180 suppressed the WT 3’-UTR activity of SCD1 and CD36, but not that of Mut 3’-UTR (Fig. 3C, D). Therefore, miR-3180 inhibited CD36 and SCD1 expression by targeting their 3’-UTRs.

### MiR-3180 inhibited lipid content in an SCD1- and CD36-dependent manner

**Fig. 4:**
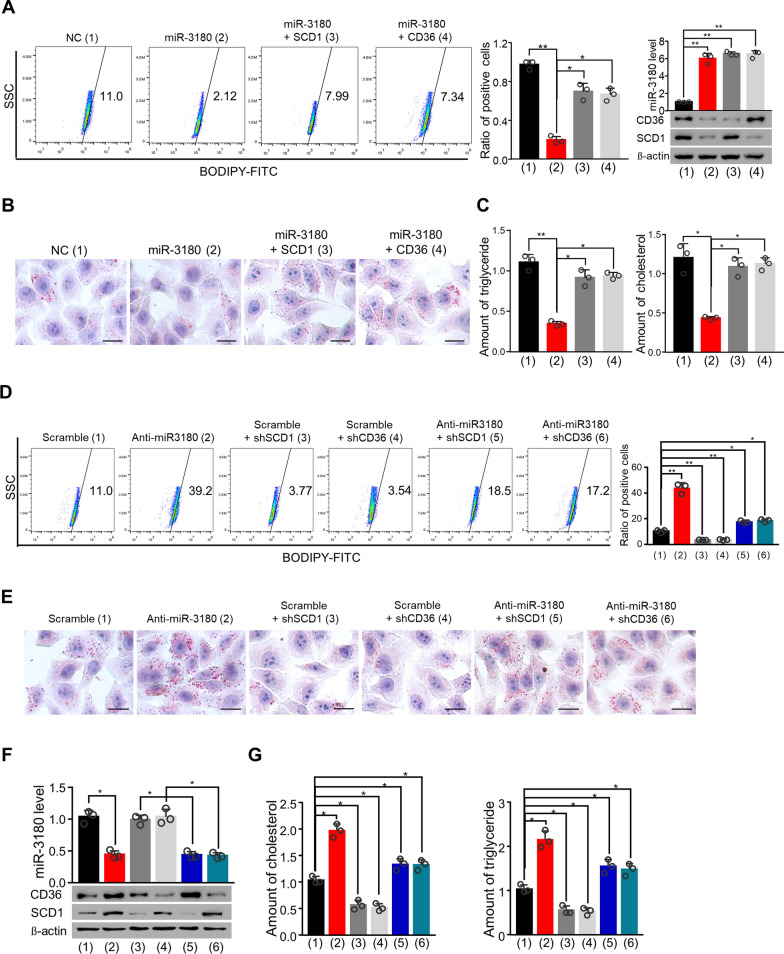
MiR-3180 suppresses lipid content in a CD36- and SCD1-dependent manner.** a** Flow cytometry analysis of lipid content in HepG2 cells transfected with miR-3180 and the indicated expression vectors or negative control. The relative lipid content is shown in the middle panel, and the expression of miR-3180, SCD1 and CD36 is shown in the right panel. **b** Oil red O staining of lipid content in HepG2 cells transfected as in (**a**). Scale bar: 20 μm. **c** The relative triglyceride and cholesterol levels in HepG2 cells transfected as in (**a**). **d** Flow cytometry analysis of lipid content in HepG2 cells transfected with the indicated vectors. The relative lipid content is shown in the right panel. **e** Oil red O staining of lipid content in HepG2 cells transfected as in (**d**). Scale bar: 20 μm. **f** RT–qPCR and immunoblotting analysis of miR-3180, CD36 and SCD1 expression in HepG2 cells transfected as in (d). G Relative triglyceride and cholesterol levels in HepG2 cells transfected as in (d). The data shown are presented as the mean ± SD of triplicate measurements with similar results. **p < 0.01

Since miR-3180 regulates lipid content and targets SCD1 and CD36, we investigated whether it regulates lipid content through these enzymes. Using flow cytometry and Oil Red O staining, we found that miR-3180 inhibited lipid content; however, SCD1 and CD36 rescued this decrease (Fig. 4A, B; Additional file 1: Fig. S1A, B). Moreover, miR-3180 reduced intracellular triglyceride and cholesterol levels, while SCD1 and CD36 overexpression reversed these effects (Fig. 4C; Additional file 1: Fig. S1C). In contrast, miR-3180 suppression by anti-miR-3180 significantly enhanced lipid content and triglyceride and cholesterol levels, and SCD1 and CD36 knockdown reversed these effects (Fig. 4D–G Additional file 1: Fig. S1D–F). In summary, miR-3180 inhibited lipid content in an SCD1- and CD36-dependent manner.

### MiR-3180 inhibited HCC cell proliferation, migration, and invasion by inhibiting SCD1 and CD36

**Fig. 5:**
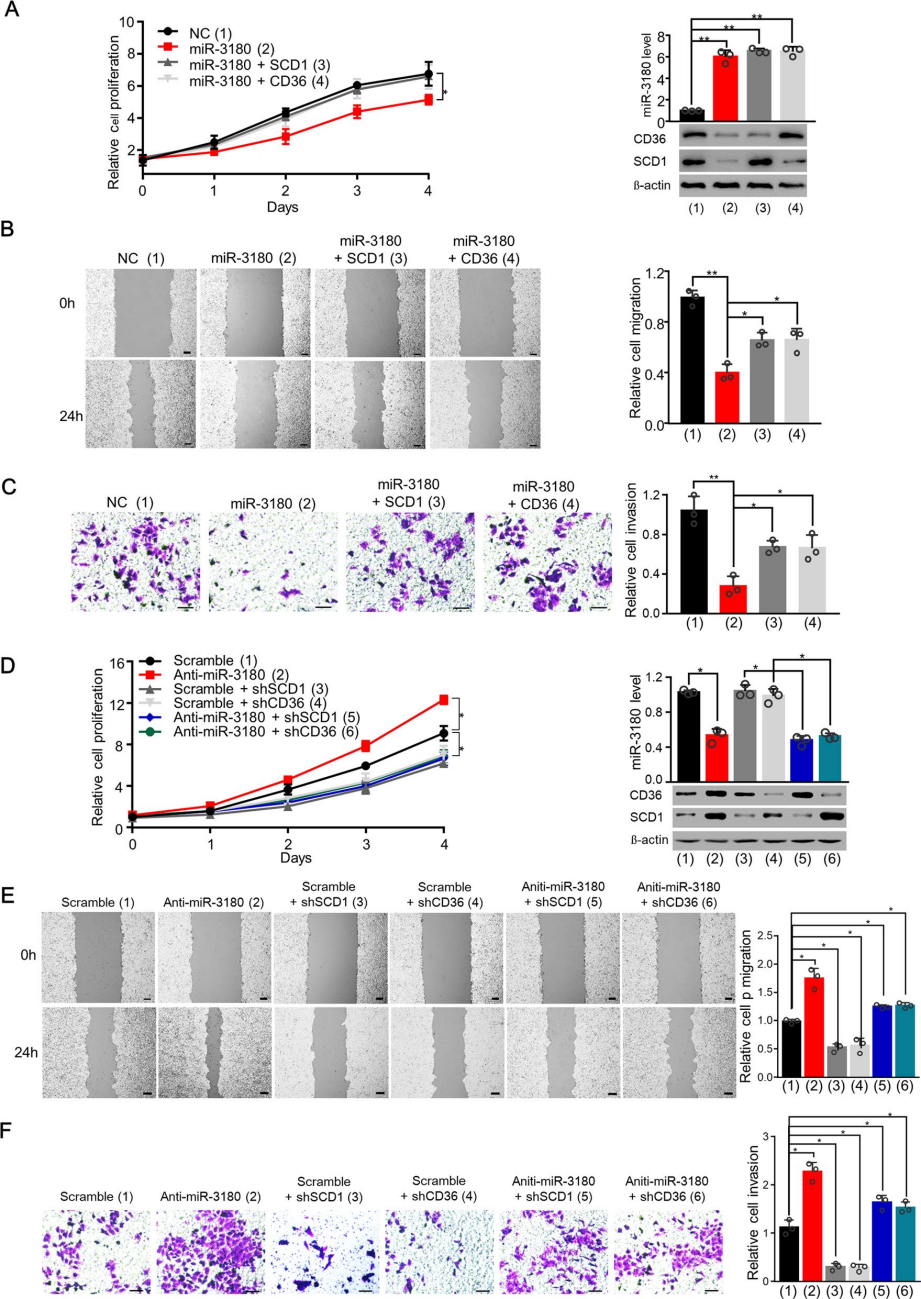
MiR-3180 inhibits the proliferation, migration, and invasion of HCC cells in a CD36- and SCD1-dependent manner. **a** Cell proliferation analysis of HepG2 cells transfected with the indicated expression vectors. The expression of miR-3180, CD36 and SCD1 is demonstrated in the right panel. **b** Wound healing analysis of HepG2 cells transfected as in (a). Scale bar: 50 μm. Statistical analysis of relative migrations is shown in the right panel. **c** Transwell analysis of HepG2 cells transfected as in (a). Scale bar: 20 μm. Statistical analysis of invasive cells is shown in the right panel. **d** Cell proliferation analysis of HepG2 cells transfected with the indicated expression vectors. The expression of miR-3180, CD36 and SCD1 is demonstrated in the right panel. **e** Wound healing analysis of HepG2 cells transfected as in (d). Scale bar: 50 μm. Statistical analysis of relative migrations is shown in the right panel. **f** Transwell analysis of HepG2 cells transfected as in (**d**). Scale bar: 20 μm. Statistical analysis of invasive cells is shown in the right panel. The data shown are presented as the mean ± SD of triplicate measurements with similar results. **p < 0.01

**Fig. 6:**
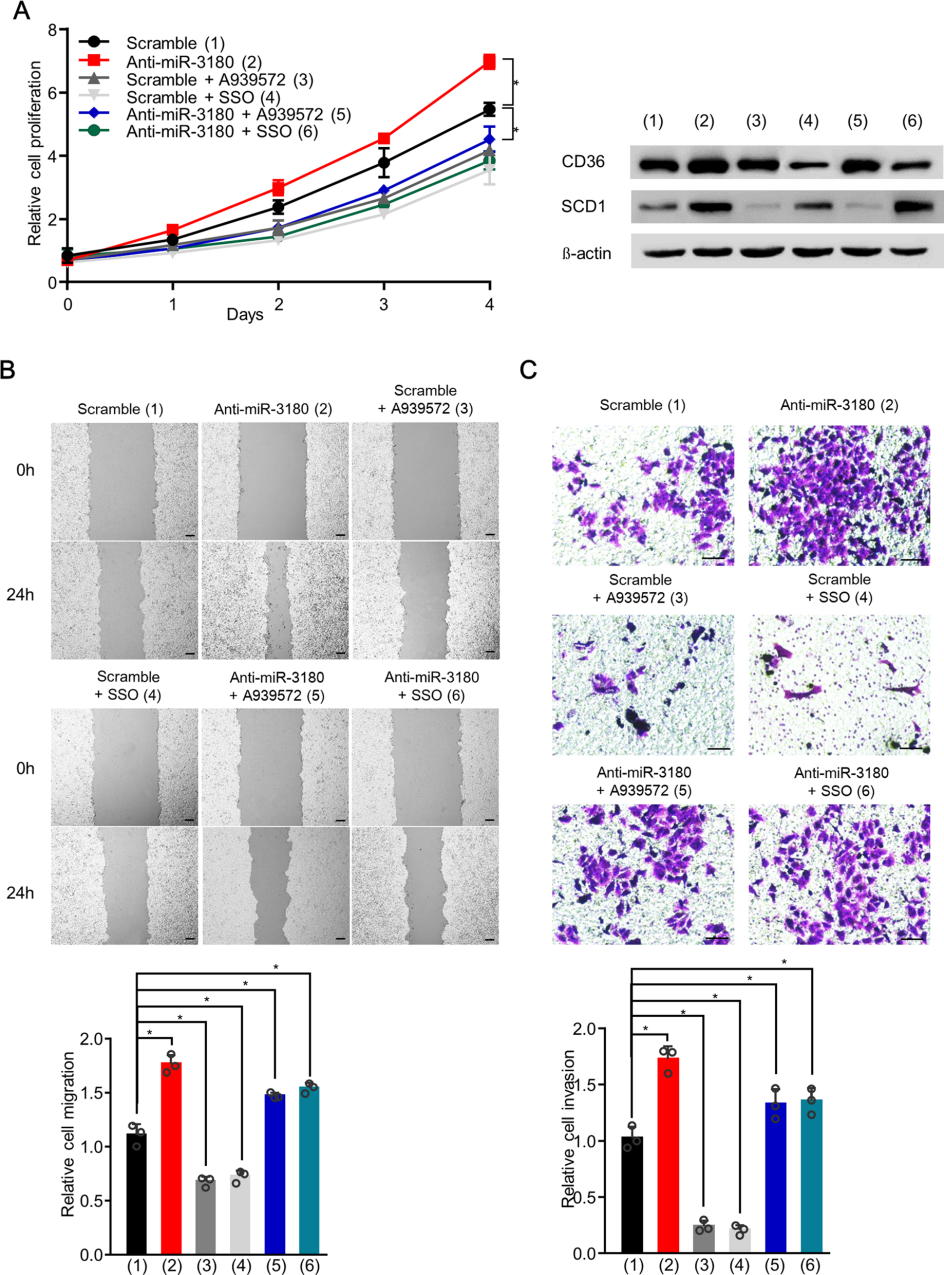
MiR-3180 inhibits the proliferation, migration, and invasion of HCC cells by suppressing lipid synthesis and uptake.** a** Cell proliferation analysis of HepG2 cells transfected with the indicated expression vectors and treated with or without A939572 (15 μM) or SSO (50 μM). The expression of CD36 and SCD1 is shown in the right panel. **b** Wound healing analysis of HepG2 cells transfected and treated as in (a). Scale bar: 50 μm. Statistical analysis of relative migrations is shown in the lower panel. **c** Transwell analysis of HepG2 cells transfected and treated as in (a). Statistical analysis of relative invasion is shown in the lower panel. Scale bar: 20 μm. The data shown are presented as the mean ± SD of triplicate measurements with similar results. **p < 0.01

Next, we investigated the effects of the miR-3180/SCD1 and miR-3180/CD36 axes on cell proliferation, migration, and invasion. MiR-3180 mimics dramatically suppressed these functions, which was reversed by SCD1 and CD36 overexpression (Fig. 5A–C; Additional file 1: Fig. S2A–C). Moreover, anti-miR-3180 enhanced cell proliferation, migration, and invasion, while SCD1 or CD36 knockdown abolished these effects (Fig. 5D–F; Additional file 1: Fig. S2D–F). In contrast, we found that the CD36 inhibitor SSO and SCD1 inhibitor A939572 abolished the anti-miR-3108-mediated enhancement in HCC cells (Fig. 6A–C; Additional file 1: Fig. S3A–C). Taken together, these data indicate that miR-3180 inhibits the proliferation, migration, and invasion of HepG2 and MHCC-97H cells through CD36 and SCD1.

### MiR-3180 suppressed tumor growth and metastasis in vivo through SCD1 and CD36

**Fig. 7:**
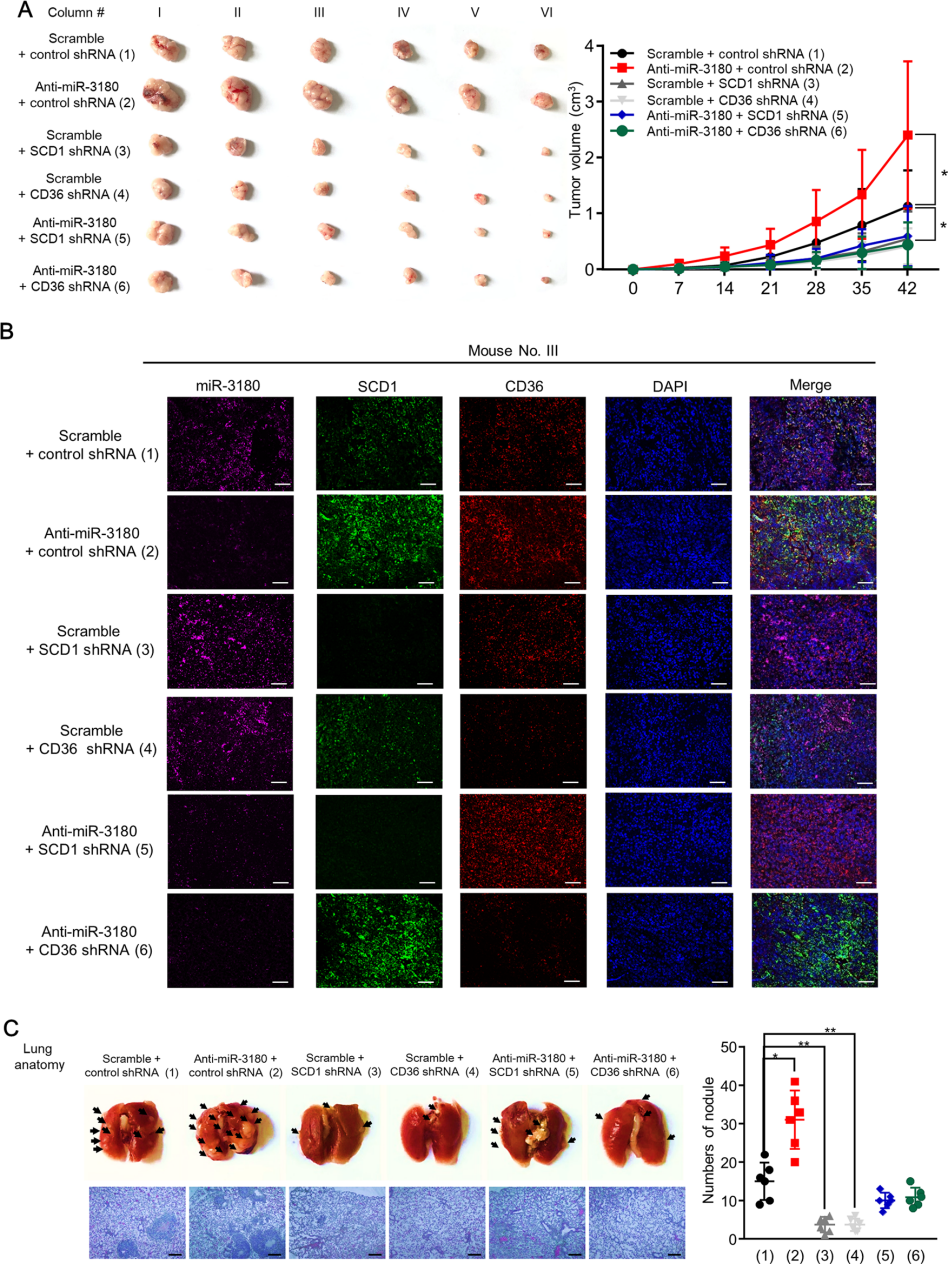
MiR-3180 represses tumor growth and metastasis in vivo by inhibiting CD36 and SCD1.** a** Volume and image of xenograft tumors of HepG2 cells infected with lentivirus carrying control shRNA, SCD1 shRNA, or CD36 shRNA and treated with miR-3180 inhibitor. The tumors were measured every 7 days with a Vernier caliper, and tumor volume curves were plotted (*n* = 6). **b** Representative immunofluorescence staining of miR-3180 (purple), SCD1 (green) and CD36 (red) in xenograft tumors from (a). **c** Representative lung tissues and H&E staining of mouse tail vein injected with MHCC-97H cells stably infected as in (a). The number of tumor nodules is presented in the right panel (*n* = 6). Scale bar: 100 μm **p < 0.01

We further investigated the effect of miR-3180 on tumor growth and metastasis in vivo using a xenograft mouse model. SCD1 and CD36 knockdown suppressed tumor growth, whereas miR-3180 inhibitor promoted it. Moreover, SCD1 and CD36 knockdown abolished anti-miR-3180-induced tumor growth, suggesting that miR-3180 regulates tumor growth through these enzymes (Fig. 7A, B).

Consistent with the in vitro data, the miR-3180 inhibitor increased triglyceride and cholesterol levels in tumor tissues, while SCD1 or CD36 knockdown inhibited this effect (Additional file 1: Fig. S4). Therefore, knockdown of SCD1 or CD36 abrogated the miR-3180 inhibitor-mediated enhancement of triglyceride and cholesterol levels.

Furthermore, the xenograft metastasis mouse model demonstrated that anti-miR-3180 promoted the lung metastasis of MHCC-97H liver cancer cells, whereas CD36 or SCD1 knockdown abrogated it (Fig. 7C). In summary, these results indicate that miR-3180 suppresses tumor growth and metastasis in vivo via SCD1 and CD36.

## Discussion

Lipid metabolism reprogramming is a hallmark of cancer development and progression [23]. At present, the lipid metabolic pathway is a target of cancer therapy. However, the regulators simultaneously involved in lipid synthesis and uptake have not been investigated. In this study, we identified this dual role of miR-3180 for the first time. First, miR-3180 expression correlated negatively with CD36 and SCD1 expression in patients with HCC. Moreover, patients with high miR-3180 expression showed longer OS than those with low miR-3180 expression. Second, miR-3180 decreased lipid content by inhibiting lipid synthesis and uptake, indicating its potential to modulate lipid metabolism. Third, miR-3180 inhibited HCC cell proliferation, migration, invasion, and metastasis through lipid synthesis and transport in vitro and in vivo, suggesting that miR-3180 is a potential target for cancer therapy through lipid metabolism.

Some studies have demonstrated the essential role of miRNAs in cancer development through lipid synthesis and uptake regulation. MiRNA-320 inhibits non-small cell lung cancer cell proliferation, migration, and invasion by targeting FASN [24]. MiR-30b-5p regulates lipid content by regulating PPAR-α [25]. MiR-342-3p suppresses uptake in the lipid uptake pathway in THP-1 cells [26]. MiR-140-5p attenuates lipid uptake and decreases intracellular cholesterol levels in HepG2 cells [27]. However, miRNAs that target both lipid synthesis and uptake have not been identified. In this study, we found that miR-3180 inhibited lipid content by suppressing both the synthesis and uptake pathways, suggesting that miR-3180 is more effective than miRNAs targeting a single pathway. Further investigation demonstrated that miR-3180 greatly suppressed HCC cell proliferation and metastasis in vivo and in vitro through lipid synthesis and uptake, suggesting that targeting both pathways is an attractive strategy for cancer therapy and presenting an attractive candidate for HCC treatment.

The controversial functions of miR-3180 in cancer have previously been reported. MiR-3180 is a biomarker of glioblastoma and colorectal cancer and is upregulated in liver metastases from patients with colorectal cancer [28]. Furthermore, miR-3180 promotes the proliferation of human bladder smooth muscle cells by targeting PODN. In contrast, it inhibits proliferation and metastasis in non-small cell lung cancer by targeting FOXP4, while the long non-coding RNA SNHG17 suppresses its tumor suppressor function in HCC [29, 30]. However, the role of miR-3180 in lipid synthesis and uptake has not been investigated yet. To the best of our knowledge, this is the first study to identify that miR-3180 affects lipid synthesis and uptake by targeting SCD1 and CD36. Moreover, we showed that miR-3180 expression correlated positively with clinical outcome, suggesting its function as an HCC biomarker.

Cancer cells acquire fatty acids via de novo synthesis or uptake from exogenous sources. Therefore, the origin of the lipids is difficult to identify. Tracing fatty acids normally uses the radioactive isotopes ^13^C or ^14^C, which causes environmental contamination and increases the risk of exposure [31, 32]. We developed a fluorescence labeling method to trace lipid metabolism [18], and found that it is sensitive and safe for lipid tracing. We found that miR-3180 inhibited CY3-labeled oleic acid transport into HCC cells. Therefore, tracing fluorescently labelled fatty acids is an effective strategy to identify the origin of lipids.

## Conclusions

In conclusion, our study identified a critical role for miR-3180 in inhibiting HCC cell proliferation, migration, invasion, and metastasis by suppressing both lipid synthesis and transport. MiR-3180 levels correlated negatively with SCD1 and CD36 expression in HCC patients. HCC patients with high miR-3180 levels showed better clinical outcomes than those with low miR-3180 levels. These data suggest that miR-3180 is a potential therapeutic target for HCC patients with high SCD1 and CD36 expression levels.

## Supplementary Information

**Additional file 1.** **Supplementary Figures.** **Figure S1.** MiR-3180 suppresses lipid content in CD36- and SCD1-dependent manner. **Figure S2.** MiR-3180 inhibits the proliferation, migration, and invasion of HCC cells in CD36- and SCD1-dependent manner. **Figure S3.** MiR-3180 inhibits the proliferation, migration, and invasion of HCC cells by suppressing lipid synthesis and uptake. **Figure S4.** The miR-3180 inhibits lipid synthesis in vivo.

## Data Availability

All data needed to evaluate the conclusions in the paper are presented in the paper and/or the Additional files. Additional data related to this paper may be requested from the authors.
