# Supplementary material for: MiR-3180 inhibits hepatocellular carcinoma growth and metastasis by targeting lipid synthesis and uptake
Source: Cancer Cell Int. 2023 Apr 11;23:66. doi: 10.1186/s12935-023-02915-9 (PMC10091558; doi:10.1186/s12935-023-02915-9)
Supplement: Supplementary file 1 — Additional file 1. Supplementary Figures. Figure S1. MiR-3180 suppresses lipid content in CD36- and SCD1-dependent manner. Figure S2. MiR-3180 inhibits the proliferation, migration, and invasion of HCC cells in CD36- and SCD1-dependent manner. Figure S3. MiR-3180 inhibits the proliferation, migration, and invasion of HCC cells by suppressing lipid synthesis and uptake. Figure S4. The miR-3180 inhibits lipid synthesis in vivo. [file 12935_2023_2915_MOESM1_ESM.pdf]

Figure S1

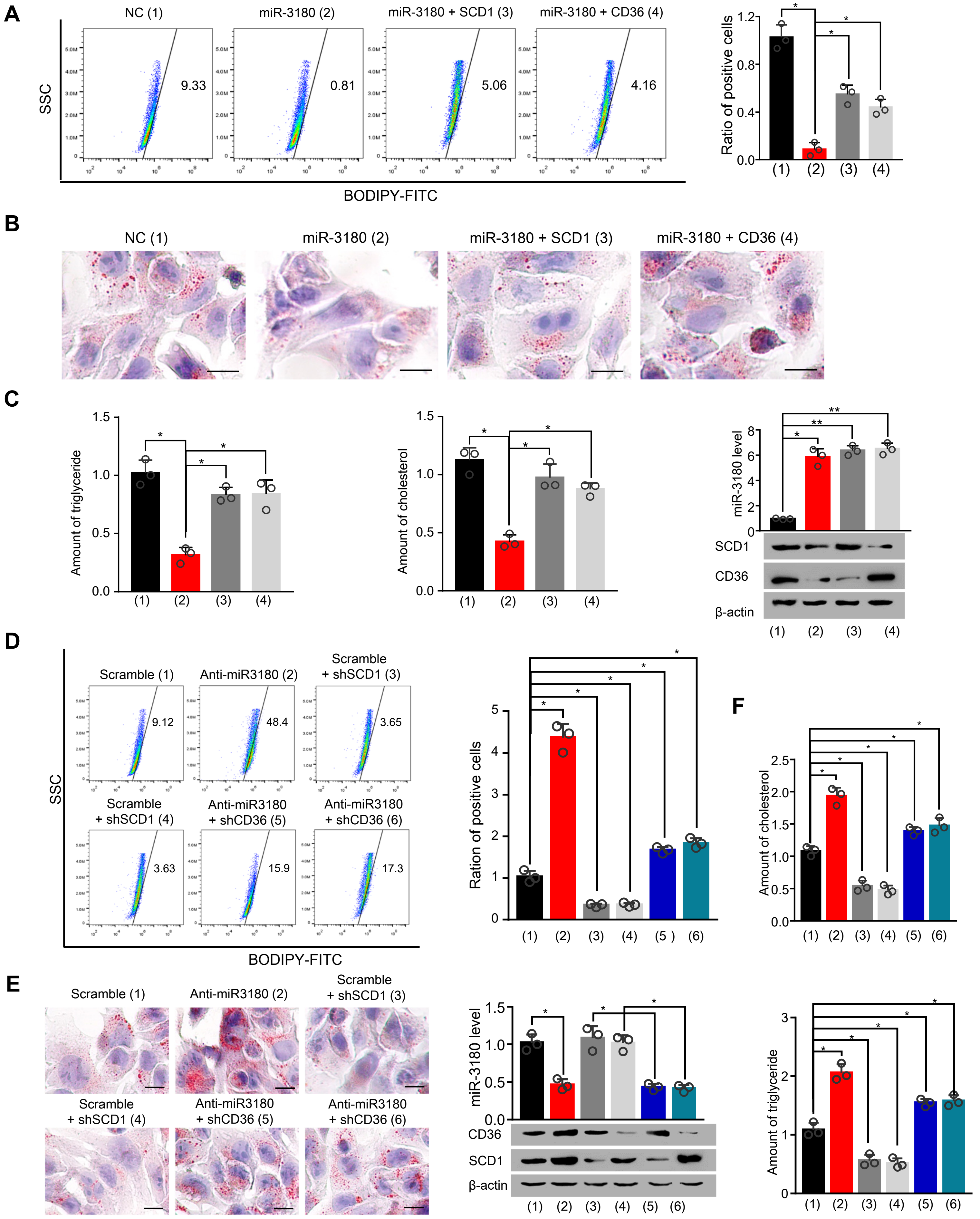

Figure S1. MiR-3180 suppresses lipid content in CD36- and SCD1-dependent manner. (**A**) Flow cytometry analysis of lipid content in MHCC-97H cells transfected with miR-3180 and the indicated expression vectors or negative control. The relative of lipid content are shown in the right panel. (**B**) Oil red O staining of lipid content in MHCC-97H cells transfected as (A). Scale bar: 20 μm. (**C**) The relative triglyceride, cholesterol and the expression of miR-3180 , SCD1 and CD36 levels in MHCC-97H cells transfected as (A). (**D**) Flow cytometry analysis of lipid content in MHCC-97H cells transfected with the indicated vectors. The relative lipid content is shown in the right nearby panel. (**E**) Oil red O staining of lipid content, RT-qPCR and immunoblotting analysis of miR-3180, CD36 and SCD1 expression in MHCC-97H cells transfected as (D). Scale bar: 20 μm. (**F**) Relative triglyceride and cholesterol level in MHCC-97H cells transfected as (D). Data shown are presented as mean ± SD of triplicate measurements with similar results. **P < 0.01.

Figure S2

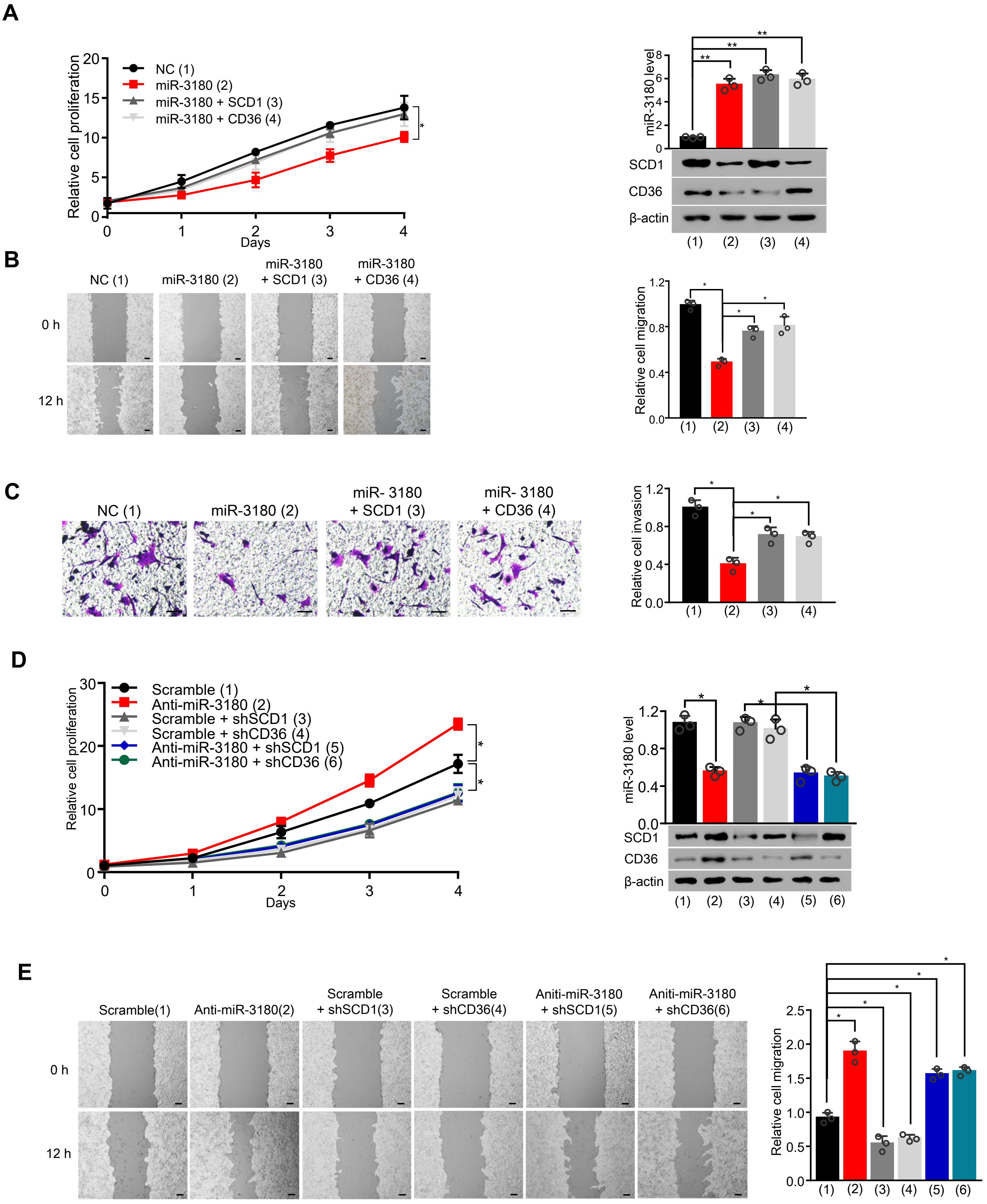
A
Relative cell proliferation
Days
NC (1)
miR-3180 (2)
miR-3180 + SCD1 (3)
miR-3180 + CD36 (4)
miR-3180 level
SCD1
CD36
β-actin
(1) (2) (3) (4)
B
NC (1)
miR-3180 (2)
miR-3180 + SCD1 (3)
miR-3180 + CD36 (4)
0 h
12 h
Relative cell migration
C
NC (1)
miR-3180 (2)
miR- 3180 + SCD1 (3)
miR- 3180 + CD36 (4)
Relative cell invasion
D
Relative cell proliferation
Days
Scramble (1)
Anti-miR-3180 (2)
Scramble + shSCD1 (3)
Scramble + shCD36 (4)
Anti-miR-3180 + shSCD1 (5)
Anti-miR-3180 + shCD36 (6)
miR-3180 level
SCD1
CD36
β-actin
(1) (2) (3) (4) (5) (6)
E
Scramble(1)
Anti-miR-3180(2)
Scramble + shSCD1(3)
Scramble + shCD36(4)
Aniti-miR-3180 + shSCD1(5)
Aniti-miR-3180 + shCD36(6)
0 h
12 h
Relative cell migration

F

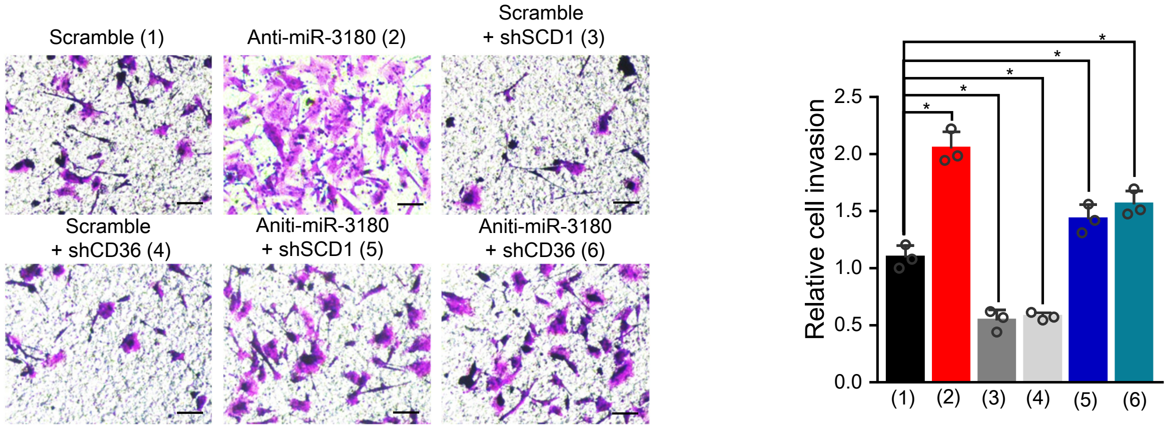

Figure S2. MiR-3180 inhibits the proliferation, migration, and invasion of HCC cells in CD36- and SCD1-dependent manner. (**A**) Cell proliferation analysis of MHCC-97H cells transfected with indicated expression vectors. The expression of miR-3180, CD36 and SCD1 are demonstrated in the right panel. (**B**) Wound healing analysis of MHCC-97H cells transfected as (A). Scale bar: 50 μm. Statistical analysis of relative migrations are demonstrated shown in the right panel. (**C**) Transwell analysis of MHCC-97H cells transfected as (A). Scale bar: 20 μm. Statistical analysis of invasion cells are shown in the right panel. (**D**) Cell proliferation analysis of MHCC-97H cells transfected with indicated expression vectors. The expression of miR-3180, CD36 and SCD1 are demonstrated in the right panel. (**E**) Wound healing analysis of MHCC-97H cells transfected as in (D). Scale bar: 50 μm. Statistical analysis of relative migrations are demonstrated shown in the right panel. (**F**) Transwell analysis of MHCC-97H cells transfected as in (D). Scale bar: 20 μm. Statistical analysis of invasion cells are shown in the right panel. Data shown are presented as mean ± SD of triplicate measurements with similar results. **P < 0.01.

Figure S3

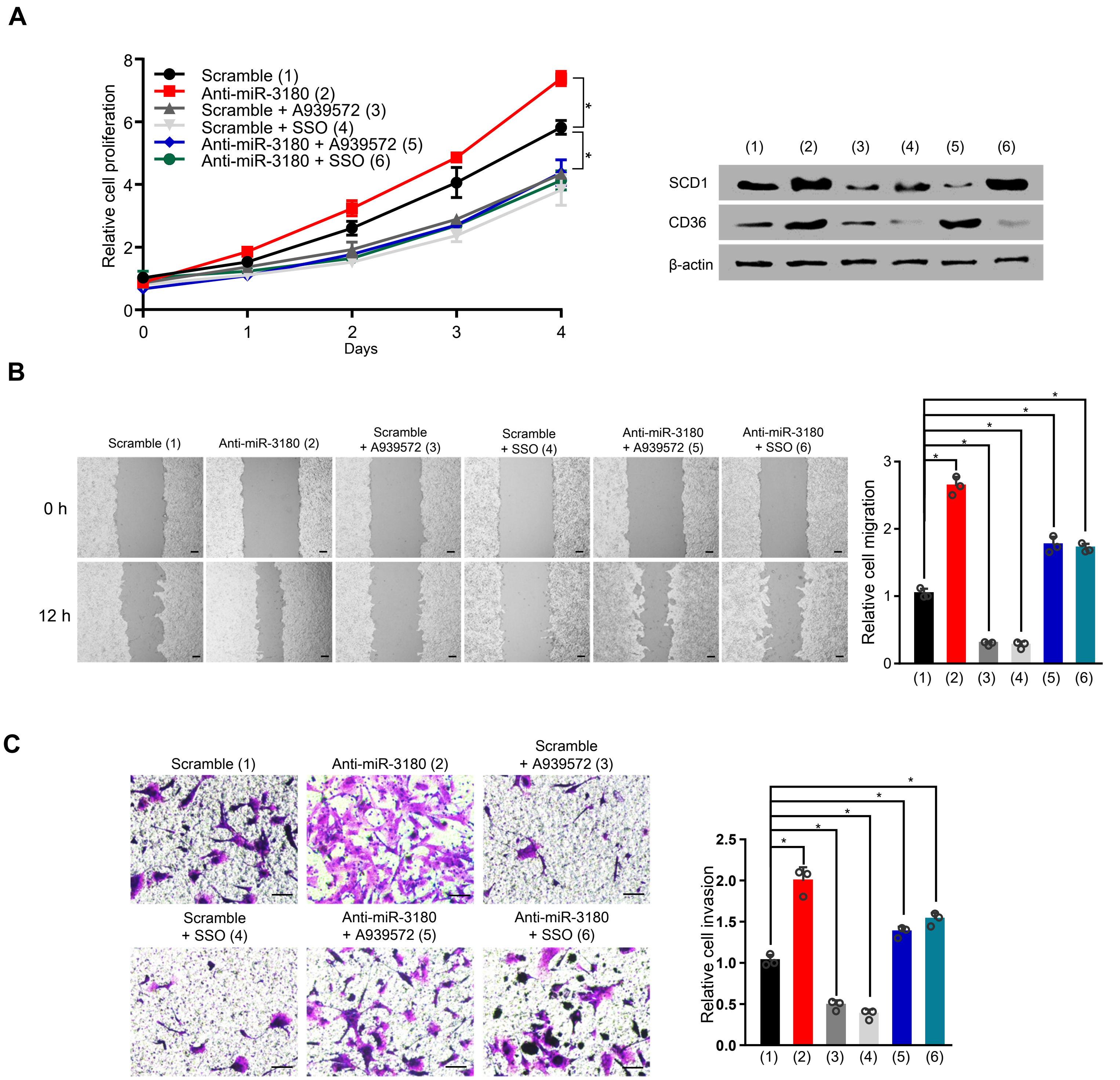

Figure S3. MiR-3180 inhibits the proliferation, migration, and invasion of HCC cells by suppressing lipid synthesis and uptake. (**A**) Cell proliferation analysis of MHCC-97H cells transfected with indicated expression vectors and treated with or without A939572 (15 µm) or SSO (50 µm). The expression of CD36 and SCD1 are shown in the right panel. (**B**) Wound healing analysis of MHCC-97H cells transfected and treated as (A). Scale bar: 50 µm. Statistical analysis of relative migrations are shown in the right panel. (**C**) Transwell analysis of MHCC-97H cells transfected and treated as (A). Statistical analysis of relative invasion are shown in the right panel. Scale bar: 20 µm. Data shown are presented as mean ± SD of triplicate measurements with similar results. **P < 0.01.

Figure S4

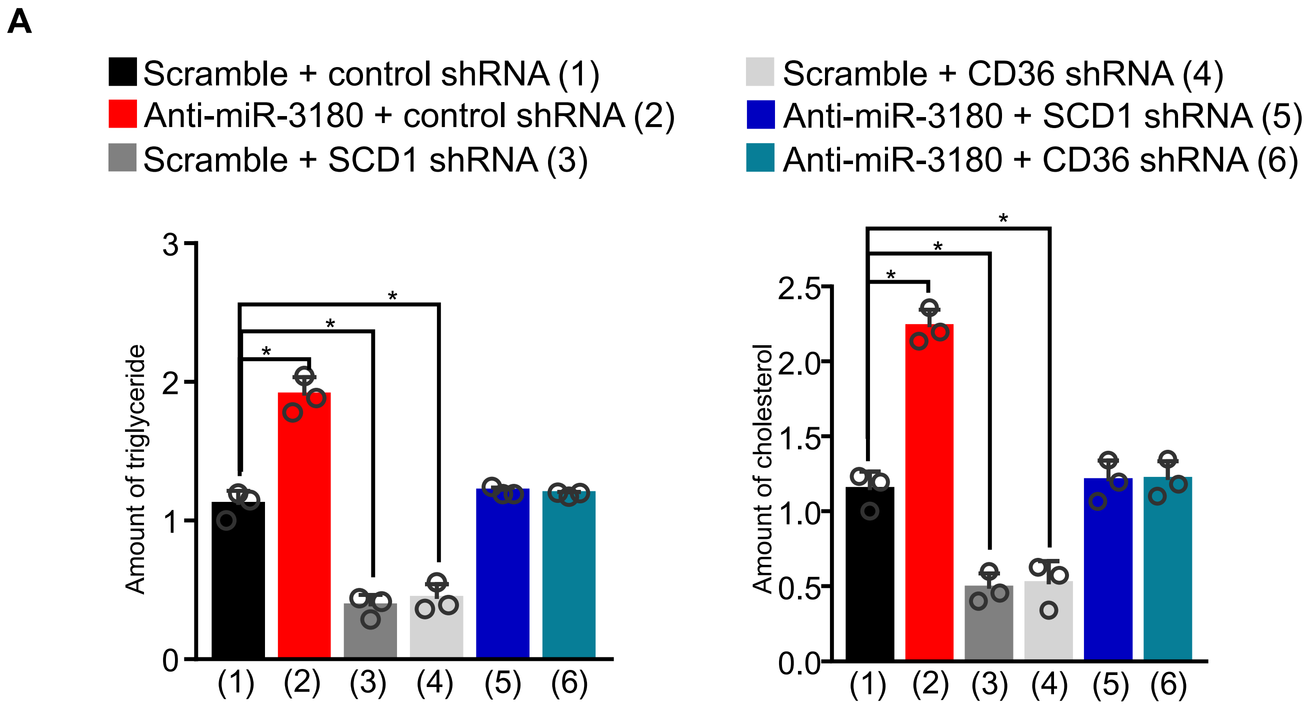

Figure S4 The miR-3180 inhibits lipid synthesis *in vivo*. Relative triglyceride and cholesterol contents in the indicated xenografts tumors from Figure 7A,B are shown.
